# Neoplastic and Non-Neoplastic Proliferative Mammary Gland Lesions in Female and Male Guinea Pigs: Histological and Immunohistochemical Characterization

**DOI:** 10.3390/ani15111573

**Published:** 2025-05-28

**Authors:** Sandra Schöniger, Claudia Schandelmaier, Heike Aupperle-Lellbach, Christina Koppel, Qian Zhang, Hans-Ulrich Schildhaus

**Affiliations:** 1 Discovery Life Sciences Biomarker Services GmbH, Germaniastrasse 7, 34119 Kassel, Germany; christina.koppel@dls.com (C.K.); hans-ulrich.schildhaus@dls.com (H.-U.S.); 2 Laboklin GmbH & Co. KG, Laboratory for Clinical Diagnostics, 97688 Bad Kissingen, Germany; schandelmaier@laboklin.com (C.S.); aupperle@laboklin.com (H.A.-L.); 3 Institute of Anatomy, Experimental Neurobiology, Goethe-University, Theodor-Stern-Kai 7, 60590 Frankfurt am Main, Germany; q.zhang@em.uni-frankfurt.de; 4 Pathology Nordhessen, Germaniastrasse 7, 34119 Kassel, Germany

**Keywords:** *Cavia porcellus*, guinea pig, pet, proliferative mammary lesions, mammary tumors, histopathology, immunohistochemistry

## Abstract

Mammary tumors occur in female and male guinea pigs. However, published data on their histology and sex predispositions are limited. Histologically, we examined proliferative mammary lesions of 69 female and 48 male pet guinea pigs. Lobular hyperplasia was observed only in females (*n* = 50). Benign tumors included simple adenomas (*n* = 20), adenolipomas (*n* = 3) and intraductal papillary adenomas (*n* = 5). All except two intraductal papillary adenomas occurred in females. Most malignancies were tubulopapillary and solid carcinomas (*n* = 54), and intraductal papillary carcinomas (*n* = 13). These were diagnosed more frequently in male (*n* = 41) than in female (*n* = 26) guinea pigs. The carcinomas of males had higher mitotic counts than those of females (*p* = 0.05). Three carcinosarcomas developed in adenolipoma, and one arose in adenoma. Results show that the mammary tumor classification of dogs and cats can be applied to guinea pigs. However, some tumors (adenolipoma, metaplastic carcinoma) are unique to guinea pigs and shared with laboratory rodents and humans, respectively. Benign tumors may undergo malignant progression. Male guinea pigs appear predisposed to ductal-associated and malignant tumors. Data suggest that male guinea pigs represent an animal model for human male breast cancer.

## Simple Summary

Guinea pigs are popular pets. Mammary tumors are reported in female and male animals. Information on their histological features and sex predispositions is limited. These data, however, are important for prognostication and medical care. By histology, this study examined proliferative mammary lesions of 117 pet guinea pigs. Lobular hyperplasia was observed only in females (*n* = 50). Benign tumors included simple adenomas (*n* = 20), benign mixed tumors (adenolipomas, *n* = 3), and intraductal papillary adenomas (*n* = 5). Except for two intraductal papillary adenomas, all benign tumors occurred in females. Most malignant tumors were simple carcinomas (*n* = 57) and intraductal papillary carcinomas (*n* = 13). These occurred more frequently in male (*n* = 42) than in female (*n* = 27) guinea pigs. The remaining malignancies of males were anaplastic carcinoma (*n* = 1) and malignant myoepithelioma (*n* = 1), and those of females, adenosquamous carcinoma (*n* = 3), carcinosarcoma in adenolipoma (*n* = 3), adenoliposarcoma (*n* = 1) and carcinoma in simple adenoma (*n* = 1). Results show that guinea pig mammary tumors include adenolipoma and its variants, benign tumors may undergo malignant progression, and males are predisposed to ductal-associated tumors and simple carcinomas.

## 1. Introduction

The domestic guinea pig (*Cavia porcellus*) is traditionally classified as a New World rodent belonging to the suborder Hystricomorpha (porcupine-like rodents) and the family Caviidae [1]. Guinea pigs have been used in medical research for over 200 years [1] and are popular pets [2]. Their usual life span is 3–4 years. However, they can reach an age of 6–7 years [1]. Boars are bred at 3–4 months of age, and sows at 2–3 months of age [1].

Female and male guinea pigs have a single pair of inguinal mammary glands in a fatty pad on either side of the abdominal midline [3]. Before puberty, in both sexes, mammary glands consist of a few dichotomously branching ducts embedded within a stroma mainly composed of adipose tissue. In males, during puberty, no further growth and even some regressive changes are initiated, so that at adulthood, their mammary tissue is composed of a primitive ductal system [3]. In contrast, at the beginning of puberty, the mammary glands of female guinea pigs show marked branching, leading to the development of ductulo-lobular units [3]. Secretory alveoli are formed during pregnancy and involute after lactation [3]. Although nipples are present in female and male guinea pigs, those of boars, however, are shorter compared to the nipples of sows [3].

So far, only a few publications on proliferative mammary lesions of guinea pigs are available. Those on pet guinea pigs represent primarily case reports [4,5], case series of 5 to 11 proliferative mammary gland lesions [6,7,8], or review of medical data [9,10,11,12]. To the authors’ knowledge, only one study examined proliferative mammary lesions in 43 pet guinea pigs [13]. In addition, mammary tumors have been investigated in laboratory guinea pigs [14,15,16]. Under consideration of all diagnosed neoplastic and non-neoplastic diseases, the reported prevalence of mammary gland neoplasms in guinea pigs is 1.3% [9]. Mammary tumors seem to occur with nearly equal frequency in female and male guinea pigs [7,11,13,16]. However, depending on the respective investigation, there might be a predominance of either female [7,11] or male animals [9,10,13].

Currently, a standardized classification for mammary tumors and non-neoplastic proliferative mammary lesions in guinea pigs has not been published. Further, the direct comparison of published proliferative mammary lesions in guinea pigs is impaired using a non-uniform diagnostic terminology [6,7,16] and a lack of tumor subclassification [6,11]. This investigation aimed to characterize mammary tumors and non-neoplastic proliferative lesions of 117 pet guinea pigs by histopathology and immunohistochemistry, classify them according to uniform criteria, and check for possible age and sex predilections.

## 2. Materials and Methods

The Laboklin GmbH and CO. KG (Bad Kissingen, Germany) database was retrospectively searched for reports describing proliferative mammary gland histological changes from 2017 to 2024. These existed for 117 guinea pigs. This study examined their proliferative mammary gland lesions by histopathology and immunohistochemistry. As all samples were routine diagnostic submissions, there was no need to submit a request for animal testing or to obtain approval from the ethics committee. This was confirmed by the decision of the local government (RUF-55.2.2-2532-1-86-5).

### 2.1. Signalment

Information on the breed, age, and sex of the 117 guinea pigs was obtained from the database.

### 2.2. Histological Processing

Tissue samples were surgical specimens that had been excised in veterinary practices, immediately fixed in 10% buffered formalin for 24–72 h, and sent to Laboklin GmbH and CO.KG. After their arrival, tissue samples were trimmed, embedded in paraffin wax, routinely processed, sectioned at 3 µm with a microtome, and stained with hematoxylin and eosin (HE). Immunohistochemistry was performed on selected cases (Table S1).

### 2.3. Histological Examination

#### 2.3.1. Evaluation of Hematoxylin and Eosin-Stained Tissue Sections

In benign and malignant tumors, the mitotic count was determined within a standard field of view of 0.237 mm^2^ as follows: within the area of the highest mitotic activity, mitotic figures were counted within ten adjacent fields of view (FOVs) at 400× magnification with an ocular FN of 22 [17,18,19] by using a Leica DM 2000 microscope (Leica Microsystems, Wetzlar, Germany). The evaluated area measured 2.37 mm^2^.

Malignant tumors were further subclassified into non-infiltrative and infiltrative neoplasms [18].

Grading of malignant tumors was based on the percentage of tubular growth in the tumor area, mitotic count, and degree of nuclear pleomorphism [18,20,21,22]. Results of each histological parameter were categorized by using a 3-tiered scoring system, which determined the tumor grade, i.e., grade I (histologic scores 3–5; well-differentiated), grade II (histologic scores 6–7; moderately differentiated), and grade III (histologic scores 8–9; poorly differentiated) [18,20,21,22]. Adenosquamous carcinomas were graded by evaluation of solely the glandular tumor area [18]. Due to a lack of tubular growth in malignant myoepithelioma, an adapted grading scheme was used [18].

#### 2.3.2. Immunohistochemistry

The Discovery Ultra staining platform (Roche Diagnostics, Tucson, AZ, USA) was used for cytokeratin AE1/AE3 immunolabeling, whereas immunolabeling for p63 and cytokeratin 14, vimentin, and α-smooth muscle actin (SMA) was performed on the Benchmark Ultra fully automated stainer (Roche Diagnostics).

In brief, after deparaffination, antigen retrieval was performed by heat incubation with the antigen retrieval solution (CC1: Roche Diagnostics). This was followed by incubation with the primary antibodies (Table 1).

Cytokeratin AE1/AE3 was detected by the sequential application of a hapten-labeled secondary antibody (anti-mouse HQ: Roche Diagnostics), an enzyme conjugate (anti-HQ HRP: Roche Diagnostics), and the chromogen. For cytokeratin AE1/AE3, 3,3′-diaminobenzidinetetrahydrochloride (DAB: Roche Diagnostics) served as chromogens. For visualization of cytokeratin 14 and p63 immunohistochemistry, the OptiView DAB detection kit (Roche Diagnostics) was used, whereas vimentin and SMA were detected with the ultraView Universal DAB detection kit (Roche Diagnostics).

After counterstaining with hematoxylin II (Roche Diagnostics), a bluing reagent (Roche Diagnostics) was applied, and slides were cover-slipped.

Normal anatomic structures present in the sections served as internal positive controls: epidermis, luminal epithelial cells, and myoepithelial cells (MECs) of the normal mammary gland for cytokeratin AE1/AE3 [7] and basal keratinocytes of the epidermis and MECs of the normal mammary gland for p63 [18,21], MECs of the normal mammary gland and sebaceous gland elements for cytokeratin 14 [18,23,24], MECs of the normal mammary gland and fibrous connective tissue for vimentin [18], and MECs of the normal mammary gland and the vascular tunica media for SMA [18]. As internal negative controls, cell types and tissues lacking expression of cytokeratin AE1/AE3, cytokeratin 14, p63,vimentin, and SMA were used. For the negative reagent control, the primary antibodies were replaced with nonimmune rabbit and mouse immunoglobulins at the appropriate concentrations.

### 2.4. Classification of Mammary Gland Proliferative Lesions

The guinea pig mammary carcinomas were classified according to the recommendations for the classification of mammary tumors in domestic animals [18]. In case observed tumors were not mentioned for dogs, cats, and other species in this fascicle [18], the nomenclature for mammary gland proliferative and nonproliferative lesions in mice and rats [25] and the World Health Organization’s classification of tumors of the human breast [26] were consulted.

### 2.5. Statistical Evaluation

The IBM SPSS software version 29 (IBM SPSS Inc., Armonk, NY, USA) was applied for statistical evaluation. To test for possible age and sex predilections, groupwise comparisons were performed by using the *t*-test (Table 2). Levene’s test was applied to measure the equality of variances between the groups. The significance threshold was set at 0.05. 

## 3. Results

### 3.1. Epidemiology

Of the 117 pet guinea pigs, 69 (59%) were female, including 67 (57%) intact and 2 (2%) spayed guinea pigs, and 48 (41%) were male, encompassing 36 (31%) intact and 12 (10%) neutered animals.

Their ages ranged from 1.5 to 9 years, with a median age of 3.5 years. Female guinea pigs had an age range between 1 and 9 years and a median age of 3.6 years. The age of male guinea pigs ranged from 1.6 to 5.9 years, with a median age of 3.7 years. The age of one male neutered animal was unknown.

Of the 117 guinea pigs, the breed was not mentioned for 84 animals (72%), 12 (10%) were named as smooth-coated, and 10 (9%) as Abyssinian. The remaining animals were Teddy (*n* = 3), long-haired (*n* = 2), Peruvian (*n* = 1), American (*n* = 1), Angora (*n* = 1), Lunkarya (*n* = 1), and crested (*n* = 1). One guinea pig was reported as a mixed breed.

### 3.2. Mammary Gland Proliferative Lesions

All 117 animals of this study had at least one proliferative mammary gland lesion; additional non-neoplastic mammary gland parenchyma was detected in 105 cases, i.e., 66 cases (63%) were from females and 39 cases (37%) from males (Figures S1–S3, Table 3).

The concurrent presence of lobular hyperplasia with secretory activity and a tumor was observed in 18 cases, i.e., adenoma (*n* = 13), adenolipoma (*n* = 1), adenoliposarcoma (*n* = 1), tubulopapillary carcinoma (*n* = 1), carcinosarcoma in adenolipoma (*n* = 1), and carcinoma in adenoma (*n* = 1). Lobular hyperplasia with fibrosis was consistently observed in association with a tumor: two were intraductal papillary adenomas, and 15 carcinomas, i.e., tubulopapillary carcinoma (*n* = 9), tubular carcinoma (*n* = 1), intraductal papillary carcinoma (*n* = 3), and adenosquamous carcinoma (*n* = 2). The simultaneous presence of lobular hyperplasia with secretory activity and lobular hyperplasia with fibrosis was always found in mammary tissue with carcinoma, i.e., tubulopapillary carcinoma (*n* = 5), solid carcinoma (*n* = 1), and intraductal papillary carcinoma (*n* = 1). Detailed information on the age distribution of animals with proliferative lesions is found in Table S2.

#### 3.2.1. Non-Neoplastic Proliferative Mammary Lesions

These were exclusively diagnosed in female guinea pigs, i.e., female intact (*n* = 49) and female-neutered (*n* = 1). Guinea pigs aged from 1 to 9 years (median: 3.63 years).

**Figure 1:**
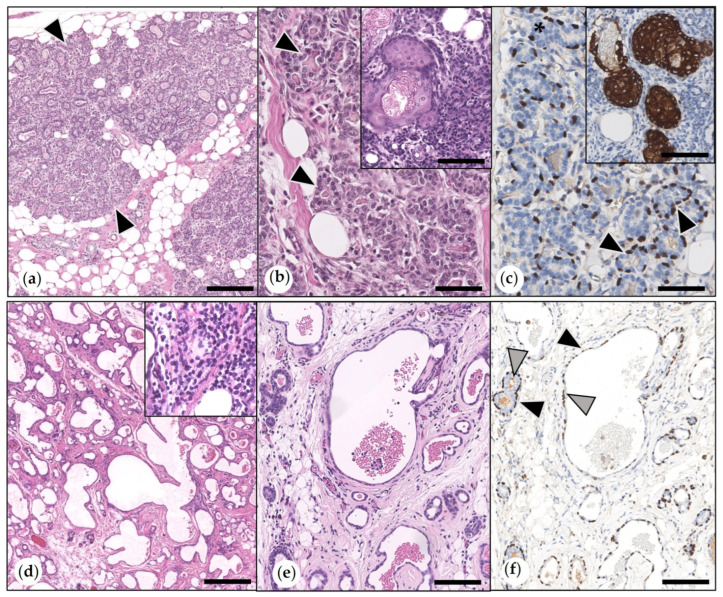
(**a**–**c**) Lobular hyperplasia with secretory activity. (**a**) Lobules were expanded by ductules and alveoli (arrowheads). Bar = 500 µm. (**b**) Depicted in higher magnification are ductular and alveolar structures (arrowheads). HE. Bar = 60 µm. Inset: intralesional sebaceous gland lobules and ducts were observed in two cases. HE. Bar = 100 µm. (**c**) p63 immunolabeling: ductules and alveoli were formed by luminal epithelial cells that were surrounded by suprabasal p63-positive myoepithelial cells (MECs, arrowheads). Bar = 60 µm. Inset: cytokeratin 14 immunohistochemistry: sebaceous glands were immunopositive (arrow). DAB. Bar = 100 µm. (**d**–**f**) Lobular hyperplasia with fibrosis. (**d**) Inter- and intralobular fibrosis with ectasia of ductules and alveoli and infiltration with mononuclear immune cells. HE. Bar = 500 µm. Inset: mononuclear immune cell infiltration is depicted in detail. HE. Bar = 60 µm. (**e**) Intralobular fibrosis with associated miniaturization and cystic dilation of ducts and alveoli. HE. Bar = 100 µm. Inset: (**f**) immunolabeling for p63: ducts and alveoli were lined by an inner layer of luminal epithelial cells (unlabeled: grey arrowheads) and an outer layer of p63-positive MECs (black arrows). DAB. Bar = 100 µm.

The most frequently observed (*n* = 26) was lobular hyperplasia with secretory activity. In one female intact animal and the female-neutered guinea pig, this lesion was associated with the presence of sebaceous gland lobules and ducts (Figure 1).

In 17 animals, there was lobular hyperplasia with fibrosis. This was characterized by inter- and intralobular fibrosis and ectasia of ductuloalveolar structures. All cases showed mild to moderate infiltration with lymphocytes, plasma cells, and occasional heterophils (Figure 1). The simultaneous presence of both types of hyperplasia was observed in seven cases.

#### 3.2.2. Benign Mammary Tumors

In 28 of 117 animals (24%), a benign mammary tumor was diagnosed. The affected guinea pigs were between 1.67 and 9 years old (median: 3 years). Notably, the majority of benign tumors (*n* = 26) were diagnosed in female intact guinea pigs. Only two intraductal papillary adenomas were detected in two male neutered animals.

- Simple Adenomas

**Figure 2:**
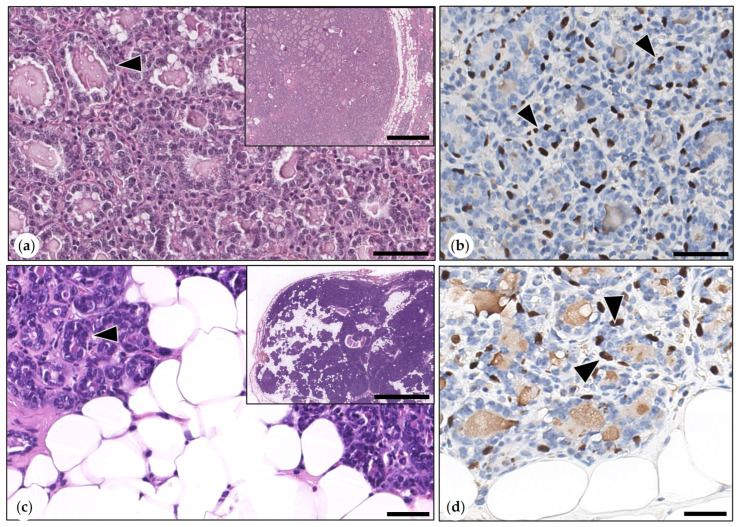
(**a**,**b**) Simple adenoma. (**a**) The tumor was composed of tubular structures (arrowheads) that were formed by tumor cells and scant stroma. HE. Bar = 100 µm. Inset: overview magnification: expansile tumor with compression of adjacent mammary tissue. HE. Bar = 500 µm. (**b**) Immunolabeling for p63: within tubular structures, neoplastic epithelial cells (unlabeled) were surrounded by a layer of suprabasal p63-positive myoepithelial cells (arrowhead). DAB. Bar = 100 µm. (**c**,**d**) Adenolipoma. (**c**) Tubular structures were delineated by epithelial tumor cells and separated by scant stroma and sheets of well-differentiated adipocytes. HE. Bar = 100 µm. Inset: the well-demarcated adenolipoma is shown at an overview magnification. HE. Bar = 1500 µm. (**d**) Immunolabeling for p63: tubular structures were lined by negative tumor cells that were surrounded by an outer layer of suprabasal p63-positive myoepithelial cells (MECs, arrowheads). DAB. Bar = 50 µm.

A tubular growth with proteinaceous secretion was present in all adenomas. Tubular structures were lined by an inner single layer of well-differentiated neoplastic epithelial cells and by an outer layer of suprabasal MECs, which were highlighted by p63 immunolabeling (Figure 2). The mitotic count of tumor cells ranged from 1 to 6 (median: 1) mitoses in 2.37 mm^2^. Four adenomas contained sebaceous gland lobules and duct-like structures. Mature sebocytes and reserve cells were cytokeratin 14-positive.

- Benign Mixed Tumors (Adenolipomas)

**Figure 3:**
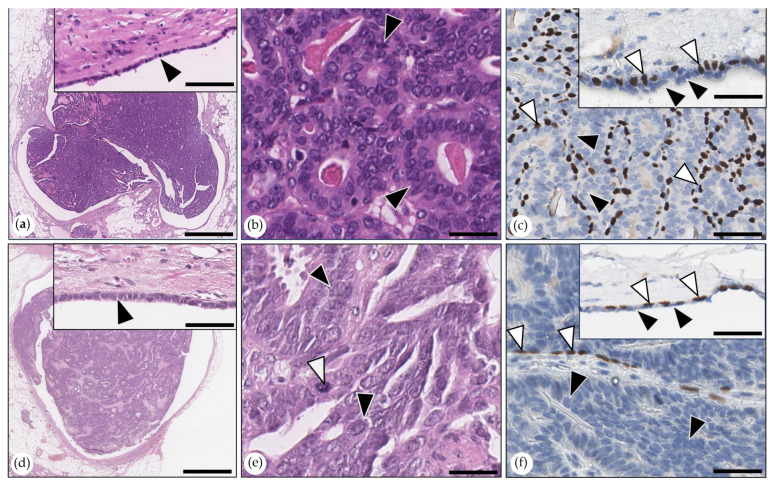
(**a**–**f**) Comparative illustration of morphological features of intraductal papillary adenoma and intraductal papillary carcinoma. (**a**–**c**). Intraductal papillary adenoma. (**a**) Benign tumor contained within a cystic duct. HE. Bar = 1500 µm. Inset: the duct wall was delineated by attenuated epithelial cells (arrowhead). HE. Bar = 50 µm. (**b**) The tumor consisted of tubulopapillary formations that were lined by one layer of monomorphic tumor cells (black arrowheads). HE. Bar = 40 µm. (**c**) p63 immunohistochemistry: negative tumor cells (black arrowheads) were surrounded by suprabasal p63-positive myoepithelial cells (MECs, white arrowheads). DAB. Bar = 50 µm. Inset: lining of the duct wall by an attenuated bilayered epithelium composed of luminal epithelial cells (unlabeled, black arrowheads) and outer p63-positive MECs (white arrowheads). DAB. Bar = 50 µm. (**d**–**f**) Intraductal papillary carcinoma. (**d**) Malignant papillary tumor contained within a cystic duct. HE. Bar = 1500 µm. Inset: the duct wall was lined by attenuated epithelial cells (arrowhead). HE. Bar = 20 µm. (**e**) The tumor consisted of tubulopapillary formations that were lined by one to several layers of slightly pleomorphic tumor cells (black arrowheads). A mitotic figure is labeled by the white arrowhead. HE. Bar = 40 µm. (**f**) p63 immunolabeling: few retained suprabasal p63-positive MECs (white arrowheads), aggregates of tumor cells (black arrowheads). DAB. Bar = 50 µm. Inset: lining of the duct wall by an attenuated bilayered epithelium composed of luminal epithelial cells (unlabeled, black arrowheads) and outer p63-positive MECs (white arrowheads). DAB. Bar = 50µm.

**Figure 4:**
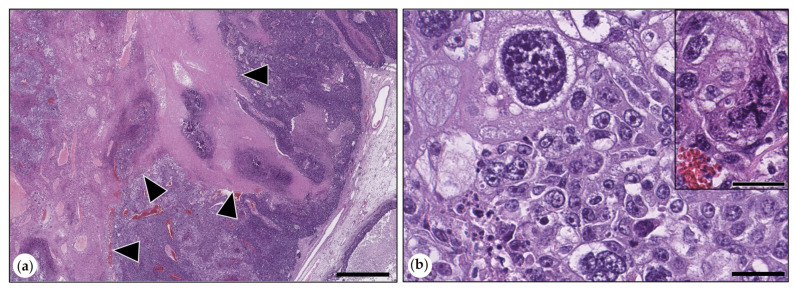
(**a**,**b**) Anaplastic carcinoma. (**a**) The tumor contained large areas of necrosis delineated by arrowheads. HE. Bar = 1000 µm. (**b**) Tumor cells were highly pleomorphic with marked anisocytosis and anisokaryosis and prominent nucleoli. HE. Bar = 50 µm. Inset: tumor cell with a mitotic figure. HE. Bar = 50 µm.

Three benign mixed tumors were consistent with adenolipomas. They were well-demarcated, expansile growing, and partially surrounded by a fibrous connective tissue capsule. Neoplastic epithelial cells formed tubular and tubulopapillary structures that were surrounded by an outer layer of suprabasal p63-positive MECs. Epithelial areas were intermingled with proliferated adipocytes (Figure 2). Mitotic figures (1–4 per 2.37 mm^2^) were solely observed in neoplastic epithelial cells.

- Intraductal Papillary Adenoma (synonym: Duct Papilloma)

The tumor was formed by an ectatic duct and intraluminal papillary projections, which were supported by stroma and lined by a bilayered epithelium. This was composed of well-differentiated cuboidal epithelial tumor cells and suprabasal p63-positive MECs. Mitotic figures were exclusively detected in epithelial tumor cells. The mitotic count varied between 1 and 4 mitoses (median: 2) (Figure 3). 

#### 3.2.3. Malignant Mammary Tumors

Malignant mammary tumors were diagnosed in 81 of 117 guinea pigs (69%).

The reported sex of guinea pigs was male intact (*n* = 36; 44%), male neutered (*n* = 10; 12%), female intact (*n* = 34; 42%), and female spayed (*n* = 1; 1%). Their ages ranged from 1.5 to 9 years (median: 4 years).

- Simple Carcinoma

Tumors included tubulopapillary carcinoma (*n* = 42), solid carcinoma (*n* = 12), tubular carcinoma (*n* = 3), and anaplastic carcinoma (*n* = 1).

The tubulopapillary, solid, and tubular histotypes were defined by a predominance of the respective growth pattern. P63 immunolabeling revealed the presence of a few retained suprabasal MECs, whereas tumor cells were immunonegative (Figure S4).

Of the tubulopapillary carcinomas, four showed ulceration through the skin (Figure S4) and five showed squamous epithelial metaplasia. The latter is defined as the presence of squamous epithelium with neoplastic features in less than 25% of the tumor area [18].

The anaplastic carcinoma was composed of islands and trabecules of highly pleomorphic tumor cells separated by thin fibrous connective tissue septae and large areas of necrosis. About 50% of tumor cells had eosinophilic cytoplasm, and the remaining tumor cells showed vacuolated to clear cytoplasm. Bizarre mitoses were observed (Figure 4). Cytokeratin AE1/AE3 immunolabeling in tumor cells ranged from marked to weak.

The mitotic count of simple carcinomas varied between 7 and 107 mitoses (median: 19) in 2.37 mm^2^. Six tumors (10%) were grade I, 46 tumors (80%) were grade II, and six tumors (10%) were grade III. Infiltrative growth was detected in 49 tumors (86%), whereas eight tumors (14%) were not infiltrative. In one tubulopapillary carcinoma, the growth in the surrounding tissue could not be determined since it was composed of tumor tissue only.

- Intraductal Papillary Carcinomas

Tumors were surrounded by an ectatic duct and consisted of intraluminal papillary proliferations that were lined by one to three layers of carcinoma cells and were supported by stroma. Multifocally, few retained suprabasal p63-positive MECs were detected. Papillary projections were partially attached to the wall of the ectatic duct, which was lined by an inner layer of epithelial cells and an outer layer of suprabasal p63-positive MECs. Tumor cells showed mild to moderate anisocytosis and anisokaryosis. The mitotic count varied between 9 and 35 (median: 23) mitoses in 2.37 mm^2^. One tumor was grade I, and the remaining tumors were grade 2 (Figure 3).

- Adenosquamous Carcinoma

**Figure 5:**
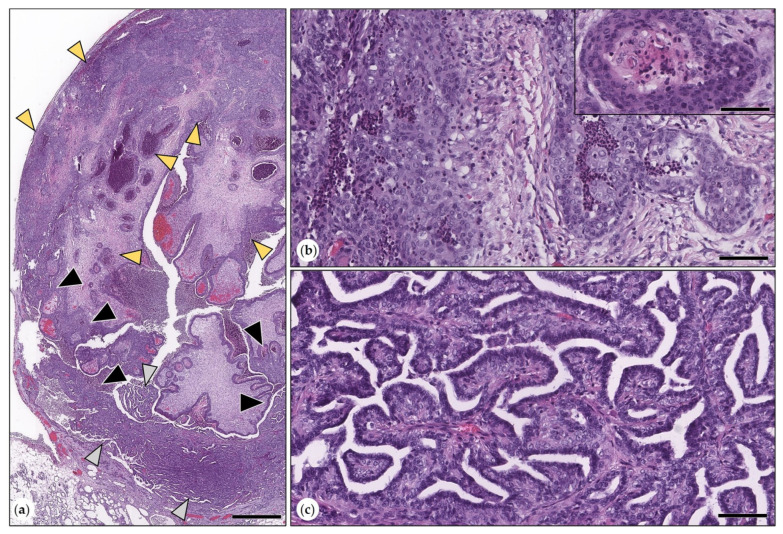
(**a**–**c**) Adenosquamous carcinoma. (**a**) Overview magnification. The tumor was composed of squamous cell carcinoma (yellow arrowheads) and (simple) tubulopapillary (adeno)carcinoma (grey arrowheads). Areas with transition between both tumor parts are labeled by black arrowheads. HE. Bar = 1000 µm. (**b**) Squamous cell carcinoma part of the tumor. Neoplastic squamous epithelial cells formed nests and islands. HE. Bar = 100 µm. Inset: tumor cell nest with central keratinization and mild acantholysis. HE. Bar = 50 µm. (**c**) Adenocarcinoma part of the tumor. Tubulopapillary proliferations were lined by neoplastic epithelial cells and supported by mild amount of stroma. HE. Bar = 50 µm.

These tumors contained intermingled areas of tubulopapillary carcinoma and squamous cell carcinoma (Figure 5). The latter encompassed about 30% of the tumor area in all three tumors. In the adenocarcinoma areas, the mitotic counts varied between 9 and 37 mitoses in 2.37 mm^2^. In the squamous cell carcinoma areas, mitotic counts varied between 3 and 16 mitoses in 2.37 mm^2^, respectively. One tumor was grade I, and two tumors were grade II. All neoplasms showed infiltrative growth.

- Malignant Myoepithelioma

**Figure 6:**
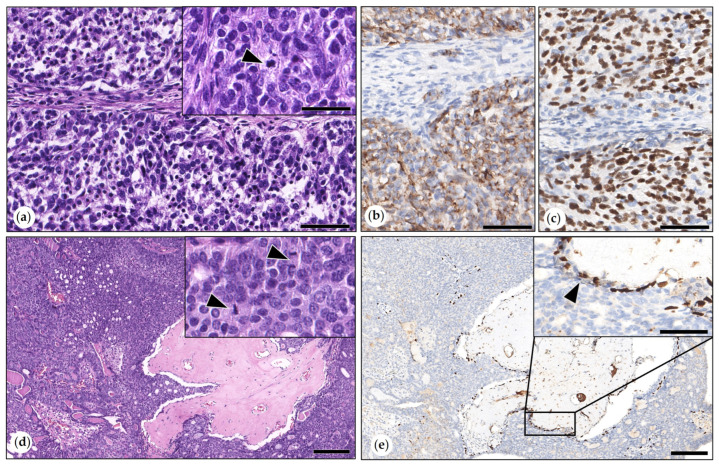
(**a**–**c**) Malignant myoepithelioma. (**a**) Aggregates of ovoid tumor cells were separated by stroma. HE. Bar = 100 µm. Inset: Tumor cells showed mild anisocytosis and anisokaryosis. Neoplastic cell with a mitotic figure (arrowhead). HE. Bar = 50 µm. (**b**) Immunolabeling for cytokeratin AE1/AE3: Neoplastic cells were positive; the stroma was negative. DAB. Bar = 100 µm. (**c**) Immunolabeling for p63: Neoplastic cells were positive; the stroma was negative. DAB. Bar = 100 µm. (**d**,**e)** Metaplastic carcinoma with mesenchymal differentiation. (**d**) Tubulopapillary carcinoma with woven bone. HE. Bar = 200 µm. Inset: densely packed tumor cells in detail, mitotic figures indicated by arrowheads. HE. Bar = 50 µm. (**e**) p63 immunolabeling: tumor cell aggregates were separated from the woven bone by a single layer of p63-positive myoepithelial cells (MECs). In the inset, the area marked by a rectangle is shown in higher magnification. DAB. Bar = 200 µm. Inset: MECs formed a single layer at the border between tumor cell nests and woven bone (arrowhead). DAB. Bar = 100 µm.

The malignant myoepithelioma was composed of ovoid tumor cells arranged in short interlacing bundles and streams. Each tumor cell had a single vesicular ovoid nucleus with one to three inconspicuous nucleoli and a scant to moderate amount of eosinophilic cytoplasm. The tumor contained numerous sebaceous gland lobules and ducts. The mitotic count was nine mitoses in 2.37 mm^2^. The tumor was infiltrative and had multifocal areas of necrosis. Tumor cells were immunopositive for cytokeratin AE1/AE3 and p63 (Figure 6).

- Carcinoma Arising in (Simple) Adenoma

**Figure 7:**
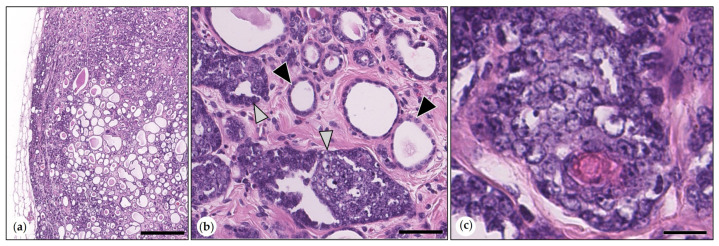
(**a**–**c**) Tubular adenoma with a multifocal carcinoma. (**a**) Tumor at an overview magnification. HE. Bar = 500 µm. (**b**) Tubular structures of the adenoma were lined by monomorphic epithelial cells (black arrowheads). Malignant neoplastic epithelial cells formed solid aggregates (grey arrowheads). HE. Bar = 50 µm. (**c**) Carcinoma in detail. Neoplastic epithelial cells had large vesicular nuclei with prominent nucleoli. HE. Bar = 20 µm.

The tumor consisted of a simple tubular adenoma containing multifocal areas of carcinoma, in which cuboidal to polygonal malignant epithelial cells form tubules and nests. These had round vesicular nuclei, moderate anisocytosis and anisokaryosis, and a mitotic count of six mitoses in 0.237 mm^2^ (Figure 7).

- Adenoliposarcoma

**Figure 8:**
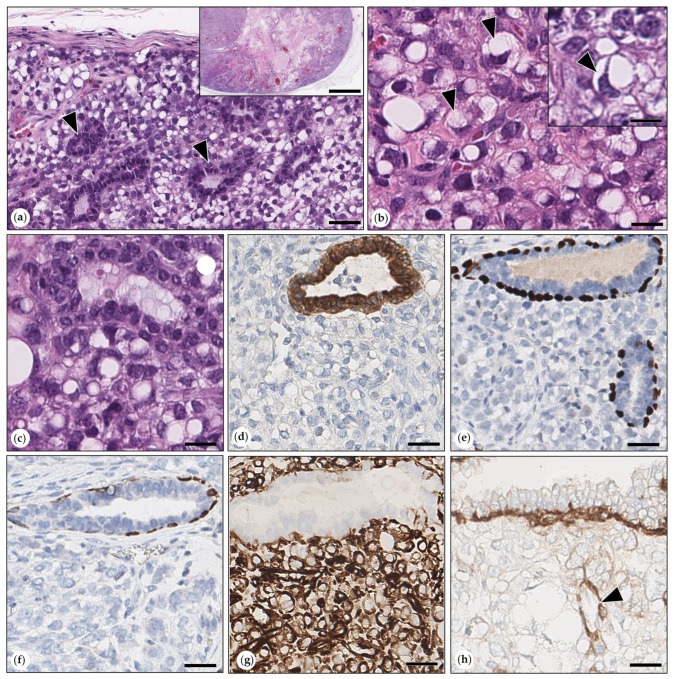
(**a**–**h**) Adenosarcoma. (**a**) The tumor consisted of well-differentiated tubular structures (black arrowheads) surrounded by sheets of pleomorphic malignant mesenchymal tumor cells, numerous of which contained variable-sized lipid vacuoles within their cytoplasm. HE. Bar = 100 µm. Inset: The well-demarcated neoplasm with a large central area of necrosis is shown at an overview magnification. HE. Bar = 1000 µm. (**b**) Malignant pleomorphic mesenchymal tumor cells are depicted in close-up. Numerous of them had large nuclei with one to several prominent nucleoli and/or one to several intracytoplasmic sharply demarcated clear vacuoles, which caused margination (arrowheads) or indentation of nuclei. HE. Bar = 50 µm. Inset: malignant neoplastic cell with two intracytoplasmic, sharply, demarcated, clear vacuoles that caused indentation of the nucleus (arrowhead). HE. Bar = 30 µm. (**c**) Tubular structures were lined by a bilayered monomorphic epithelium. HE. Bar = 30 µm. (**d**) Immunolabeling for cytokeratin AE1/AE3. Epithelial cells lining tubular structures were cytokeratin AE1/AE3 positive. Pleomorphic malignant tumor cells were cytokeratin AE1/AE3-negative. DAB. Bar = 50 µm. (**e**) Immunolabeling for p63: Within tubular structures, the inner layer of epithelial cells was immunonegative, whereas the outer layer was p63. Pleomorphic malignant tumor cells were p63-negative. DAB. Bar = 50 µm. (**f**) Immunolabeling for cytokeratin 14: Within tubular structures, the inner layer of epithelial cells was immunonegative, whereas the outer layer was cytokeratin 14-positive. Pleomorphic malignant tumor cells were cytokeratin 14-negative. DAB. Bar = 50 µm. (**g**) Immunolabeling for vimentin: Within tubular structures, the inner layer of epithelial cells was immunonegative, whereas the outer layer was vimentin-positive. Pleomorphic malignant tumor cells were vimentin-positive. DAB. Bar = 50 µm. (**h**) Immunolabeling for α-smooth muscle actin (SMA): within tubular structures, the inner layer of epithelial cells was immunonegative, whereas the outer layer was SMA-positive. Pleomorphic malignant tumor cells were SMA-negative. A vascular structure with SMA-positive tunica media is labeled by the arrowhead. DAB. Bar = 50 µm.

This expansile growing neoplasm was surrounded by a thin fibrous connective tissue capsule and had a large central area of necrosis. It was composed of tubular structures surrounded by sheets of moderately pleomorphic tumor cells. Tubular structures were lined by monomorphic neoplastic cytokeratin AE1/AE3-positive epithelial cells with three mitotic figures in 2.37 mm^2^, which were delineated by a single layer of cytokeratin AE1/AE3 and p63, vimentin, and SMA-positive suprabasal MECs. Intervening neoplastic cells were plump, spindle-shaped, polygonal, or round. Each cell had a single round vesicular nucleus with one to several prominent nucleoli. The cytoplasm of numerous cells contained one to several sharply demarcated clear vacuoles that often resulted in the indentation or margination of the nucleus. The mitotic count was five mitoses within 2.37mm^2^. These cells were positive for vimentin and negative for SMA. They were also immunonegative for cytokeratin AE1/AE2, cytokeratin 14, and p63 (Figure 8). In addition, the tumor contained scattered lobules of sebaceous glands that were positive for cytokeratin 14.

- Carcinosarcomas in Benign Mixed Tumors (Adenolipoma)

These three well-demarcated and expansile growing tumors consisted of retained lipoadenoma components, multifocal areas of sarcoma and carcinoma, and areas of necrosis. One tumor contained a few sebaceous gland lobules and ducts.

Sarcomatous differentiation was characterized by bundles of ovoid to spindle-shaped tumor cells embedded in a moderate amount of eosinophilic extracellular matrix. Neoplastic cells were positive for vimentin and negative for cytokeratin AE1/AE3, p63, cytokeratin 14, and SMA. They displayed moderate anisocytosis and anisokaryosis. Mitotic counts were 6, 6, and 10 mitoses in 2.37 mm^2^, respectively. 

**Figure 9:**
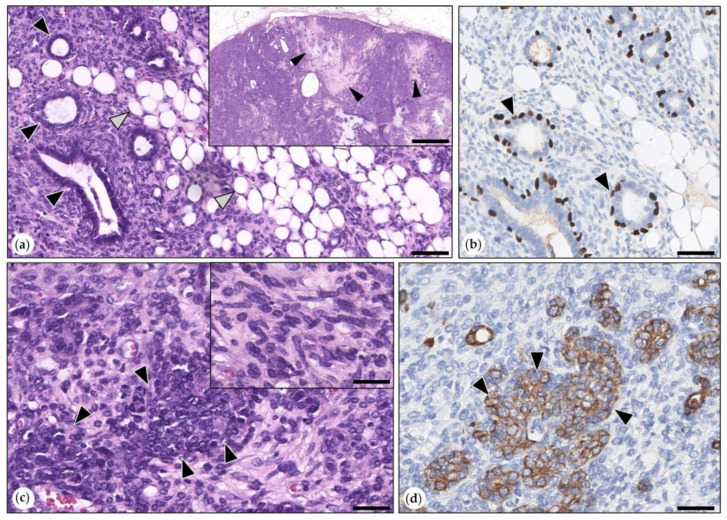
(**a**–**d**) Carcinosarcoma in benign mixed tumor (adenolipoma). (**a**) Sheets of mature adipocytes (grey arrowheads) and well-differentiated tubular structures (black arrowheads) represented retained areas of adenolipoma. HE. Bar = 100 µm. Inset: the tumor is shown at an overview magnification. It was well-demarcated, expansile growing, and contained multifocal areas of necrosis (arrowheads). HE. Bar = 1000 µm. (**b**) Immunolabeling for p63: tubular structures were lined by an inner layer of monomorphic negative epithelium delineated by p63-positive suprabasal myoepithelial cells (black arrowheads). DAB. Bar = 100 µm. (**c**) The tumor contained multifocal areas of carcinoma (black arrowheads) and sarcoma. HE. Bar = 50 µm. Inset: areas of sarcoma with pleomorphic malignant tumor cells are depicted in detail. HE. Bar = 30 µm. (**d**) Immunolabeling for cytokeratin AE1/AE3: areas of carcinoma (arrowheads) are highlighted, whereas the intermingled sarcomatous part remained unlabeled. DAB. Bar = 50 µm.

Carcinomatous differentiation was characterized by islands and trabecules of polygonal cytokeratin AE1/AE3-positive and p63-, cytokeratin 14-negative, SMA-negative, and vimentin-negative tumor cells with moderate anisocytosis and anisokaryosis. Carcinoma cells showed 8, 11, and 11 mitoses in 2.37 mm^2^ (Figure 9).

- Metaplastic Carcinomas with Mesenchymal Differentiation

These two infiltrative carcinomas showed woven bone formation devoid of malignancy features, surrounded by carcinoma cells without intervening spindle to stellate interstitial MECs or stroma tissue. One tumor was a solid carcinoma with 10 mitoses in 2.37 mm^2^, and the other tumor was a tubulopapillary carcinoma with 20 mitoses in 2.37 mm^2^. Both tumors were grade II. P63 immunolabeling revealed retained suprabasal MECs at the periphery of tumor cell nests (Figure 6). One tumor showed ulceration through the skin.

### 3.3. Results of Statistical Evaluation

No statistically significant age differences were detected between female guinea pigs with solely lobular hyperplasia, a benign tumor in the presence or absence of lobular hyperplasia, or a malignant tumor with and without concurrent lobular hyperplasia.

Regarding carcinomas diagnosed in more than three animals, i.e., intraductal papillary, tubulopapillary, and solid carcinomas, those of male guinea pigs showed higher mitotic counts (*p* = 0.05) than those of female animals. Carcinomas of male neutered animals had higher mitotic counts (*p* = 0.047) than those of male intact guinea pigs. Moreover, carcinomas of male neutered guinea pigs showed less often infiltrative growth than those of female intact (*p* = 0.003) and male intact guinea pigs (*p* = 0.010) (Table 4).

The separate analysis of carcinoma subgroups, i.e., intraductal papillary carcinomas and tubulopapillary carcinomas, revealed significantly higher mitotic counts (*p* = 0.002) in tubulopapillary carcinomas of male guinea pigs compared to those of female guinea pigs.

All other comparisons (Table 2) did not show statistically significant results; in particular, no statistically significant age differences were detected between male and female animals with the examined carcinomas and their subtypes.

## 4. Discussion

In the present study, 56% of mammary tumors occurred in female and 44% in male guinea pigs, which confirms the high incidence of mammary tumors in male guinea pigs. This is the opposite situation in companion animals [27,28,29] and human beings [26,30]. For example, mammary tumors in intact female dogs and cats account for 42% and 25% of all tumors, respectively [27]. In contrast, in male dogs and cats their incidence is 1–5% [31,32]. With up to 24% of all female cancers, breast cancer is the most frequently diagnosed malignant tumor in women [26], whereas male breast cancer occurs with a frequency of less than 1% [26,30]. Currently, the cause for the relatively high incidence of mammary tumors in male guinea pigs is uncertain.

In this study, we classified proliferative mammary lesions in guinea pigs based on the histological classification of mammary tumors of domestic animals [18]. This is consistent with previous investigations on mammary tumors of pet guinea pigs [7,8]. Only for tumors not described in this fascicle, the classification for mammary tumors in laboratory mice and rats [25] or the WHO classification for tumors in human beings [26] was consulted. The rationale for this stepwise classification approach was that all guinea pigs in this study were pet animals. Laboratory and pet animals may develop other types of tumors due to differences in housing, life expectancy, and genetics, and phylogenetic distances exist between guinea pigs, mice, and rats. Although traditionally, guinea pigs have been placed in a different rodent suborder than mice and rats, i.e., hystricomorpha, they may even constitute a separate mammalian order distinct from rodents [33,34]. The results of this study indicate that the extrapolation of a mammary tumor classification scheme from one species to another may not be adequate.

The diagnosis of the majority of proliferative mammary gland lesions can be reached by an examination of an HE-stained tissue section [18]. Immunohistochemistry is mainly applied to visualize non-neoplastic MECs and to detect epithelial, myoepithelial, and mesenchymal components in mixed tumors and those with spindle-shaped or pleomorphic tumor cells [18]. In this study, we used cytokeratin AE1/AE3 and p63. Epithelial (tumor) cells are recognized by cytokeratin AE1/AE3 expression, (neoplastic) MECs show immunolabeling for cytokeratin AE1/AE3 [7,35] and p63 [7,35], and (neoplastic) mesenchymal cells are negative for these markers [35].

Most diagnosed proliferative mammary gland lesions of guinea pigs were described in the classification of mammary tumors in domestic animals [18]. Exceptions were different types of mixed tumors, i.e., adenolipoma, carcinosarcoma in adenolipoma, adenoliposarcoma, and metaplastic carcinoma with mesenchymal differentiation.

In this study, lobular hyperplasia was the only non-neoplastic proliferative lesion. We diagnosed it solely in female guinea pigs. It was commonly associated with a tumor, most of which were malignant. Our findings are consistent with the literature data. Hyperplastic mammary lesions are mostly described in the mammary tissue of female guinea pigs with a concurrent tumor [13], and most of these tumors represent carcinomas [13]. They have only been reported in two male intact pet guinea pigs [10,13]; no information about possible predisposing conditions was provided [10]. In male animals [18] and male humans [26,36], lobular hyperplasia is commonly associated with hormonal imbalances, specifically hyperestrogenism [18,26,36]. Further investigations are necessary to determine if lobular hyperplasia, in particular lobular hyperplasia with fibrosis, could represent a premalignant change in the guinea pig.

Of the 28 benign mammary tumors of this study, all tumors, except two intraductal papillary adenomas, were detected in female guinea pigs. This is comparable to literature data, i.e., simple and tubular adenomas, as adenolipomas were described only in female guinea pigs [6,7,13]. A possible explanation would be a hormonal influence. Ovarian cysts are observed in more than 60% of adult female guinea pigs [37]. The suspected causes are hormonal imbalances, and some types of cysts may lead to elevated estrogen levels [37]. In the literature, simple tubulopapillary and solid carcinomas, as well as intraductal papillary carcinomas, were reported in female and male guinea pigs [7,13,16]. This is in agreement with our results from this study. In another investigation, ductal carcinomas were diagnosed as the most frequent malignant mammary tumors in pet guinea pigs, and most of these tumors were observed in males [13].

Male guinea pigs appear to be predisposed to mammary tumors that develop from the neoplastic transformation of ductal structures. We found benign tumors in male guinea pigs restricted to the ducts, as previously described [7,13]. In addition, we observed a high frequency of intraductal papillary carcinomas in male guinea pigs, which confirms the results of two other investigations [13,16], one of which detected a high incidence of ductal carcinomas in male guinea pigs as well [13]. It is reported that older guinea pigs are more likely to have a malignant tumor than younger guinea pigs [11]. In this investigation, we found no statistically significant age differences for female guinea pigs with non-neoplastic proliferative lesions, benign or malignant tumors, or between male and female animals with carcinomas and their subtypes. Our study revealed significantly higher mitotic counts in carcinomas and tubulopapillary carcinomas of male guinea pigs compared to those of female animals. This suggests that carcinomas of male guinea pigs may show faster growth than those of female guinea pigs. Significantly higher numbers of infiltrative carcinomas in male intact and female intact guinea pigs compared to male neutered animals implicate that the neutering status could also influence the biological behavior of mammary carcinomas.

This study also contained an adenosarcoma. This tumor was composed of benign epithelial cells forming tubular structures and intervening pleomorphic malignant mesenchymal tumor cells. The bilayered cytokeratin AE1/AE3-positive tubular epithelium consisted of an inner layer of luminal epithelial cells and an outer MEC layer that was positive for p63, CK14, vimentin, and SMA. Malignant tumor cells were identified as mesenchymal cells based on their negative immunolabeling for cytokeratin A1/AE3 and their positive vimentin immunoreaction, together with the lack of expression of the MEC markers cytokeratin 14 and p63. The negative labeling for SMA confirmed a lack of myoepithelial differentiation and ruled out myofibroblastic and smooth muscle differentiation. Numerous mesenchymal cells contained one to several intracytoplasmic clear vacuoles with distinct margins that indented the nucleus and/or resulted in the margination of the nuclei resembling mature and immature adipocytes and lipoblasts. However, this retrospective study used FFPE material, which precluded the application of a lipid stain, such as a Sudan red stain, to confirm the presence of lipid droplets in the malignant tumor cells. 

To our knowledge, adenosarcoma has not been previously published in the mammary glands of animals or the human breast. However, malignant phyllodes tumor of the human breast is composed of a benign epithelial component and a malignant mesenchymal component that may show lipomatous differentiation [26]. In human beings, adenosarcomas occur as rare tumors in the uterus or ovary [38]. In addition, three adenolipomas contained multifocal areas of carcinoma and sarcoma development. To our knowledge, this is the first description of the malignant progression of adenolipomas in guinea pigs, other companion animals, and humans. Since it was detected simultaneously within both tumor components, these tumors were diagnosed as carcinosarcoma in adenolipoma. Carcinomas arising in benign mixed mammary tumors are common in bitches [18]. Further, adenocarcinomas and sarcomas arising in mammary fibroadenomas are reported in laboratory rats and mice [25]. Two carcinomas of this study contained well-differentiated woven bone but lacked spindle-shaped to stellate interstitial MECs. Therefore, these tumors did not fulfill the definition of mixed carcinomas [18]. They were diagnosed as metaplastic carcinomas with mesenchymal elements [26] since their morphological features were identical to those of mammary tumors of humans [26]. 

Results of our study lead to the recommendation of early surgical excision of mammary tumors in female and male guinea pigs because, in females, benign mammary tumors may undergo malignant progression, and male guinea pigs are predisposed to malignant tumors.

Follow-up data were not available for the guinea pigs of this study. The performed tumor classification, however, provides the basis for further investigations into the prognosis and survival of guinea pigs with mammary tumors.

Whereas numerous induced and spontaneous animal models are available for breast cancer in women [39], currently, no animal model exists for male breast cancer [40]. The results of this investigation would support the likely value of male guinea pigs as animal models for spontaneous male breast cancer in several aspects. Notably, the tubulopapillary and solid carcinomas diagnosed in guinea pigs of this study are histologically comparable to human invasive breast cancer of non-specific type [26], which is, with 83%, the most prevalent type of male breast cancer [36,40]. Another less common tumor of the male breast is the intraductal papillary carcinoma [40]. In this study, all but three carcinomas of male guinea pigs represented tubulopapillary carcinoma, solid carcinoma, or intraductal papillary carcinoma.

It has been shown that the majority of male breast cancer cases express hormone receptors and thus belong to the molecular subtypes luminal A or luminal B [26,40,41]. So far, immunolabeling for estrogen and progesterone receptors has only been reported in two simple tubulopapillary mammary carcinomas of male guinea pigs that displayed the detection of receptors in nearly all tumor cells [7]. This study’s morphological classification of mammary tumors in guinea pigs provides the basis for molecular subclassification of simple carcinomas and, thus, for further comparative investigations into the value of male guinea pigs as animal models for male breast cancer.

## 5. Conclusions

In this study, we classified proliferative mammary lesions of guinea pigs according to standardized guidelines. The results show that the mammary tumor classification scheme adapted to dogs and cats can be applied to guinea pigs as well. However, some tumors (adenolipoma and their variants as well as metaplastic carcinoma) are unique for this species and shared with laboratory rodents and humans, respectively. We further confirmed the high incidence of mammary tumors in male guinea pigs and revealed some unique features of proliferative mammary gland lesions in guinea pigs, which were partially gender-specific. We detected that male guinea pigs have a predisposition for ductal-derived mammary neoplasms and malignant mammary tumors. Our results also indicated possible differences in the biological behavior of mammary carcinomas between male and female guinea pigs. Further, they showed that benign tumors may undergo malignant progression. Further, our data support the hypothesis that male guinea pigs could possibly represent suitable animal models for male breast cancer.

## Figures and Tables

**Table 1:** Summary of performed immunolabeling.

Primary Antibody					Antigen Retrieval
Antigen	Clone	Source	Dilution	ICT	Buffer/ICT
PanCK	AE1/AE3	Mouse	RTU	60 min	CC1/48 min
p63	4A4	Mouse	1/50	20 min	CC1/64 min
CK 14	SP53	Rabbit	1/200	16 min	CC1/32 min
Vim	V9	Mouse	RTU	16 min	CC1/36min
SMA	EP188	Rabbit	1/100	32 min	none

CK = cytokeratin; Vim = vimentin; SMA = α-smooth muscle actin; ICT = incubation time; RTU = ready to use; min = minutes.

**Table 2:** Statistical evaluations.

Group 1			Group 2			Analyzed Parameter(s)
Sex	Lesion	N	Sex	Lesion	N	
FI, FS	Hyperplasia ^1^	8	FI, FS	Benign tumor ^2^	26	Age
FI, FS	Hyperplasia ^1^	8	FI, FS	Malignant tumor ^3^	35	Age
FI, FS	Benign tumor ^2^	26	FI, FS	Malignant tumor ^3^	35	Age
FI, FS	(TPC, SC, IPC) ^4^	26	MI, MN	(TPC, SC, IPC) ^4^	41	Age, mitotic count, tumor grade
FI, FS	(TPC, SC, IPC) ^4^	26	MI, MN	(TPC, SC, IPC) ^4^	40	Infiltrative growth
MI	(TPC, SC, IPC) ^4^	31	MN	(TPC, SC, IPC) ^4^	10	Age, mitotic count, tumor grade
MI	(TPC, SC, IPC) ^4^	30	MN	(TPC, SC, IPC) ^4^	10	Infiltrative growth
FI	(TPC, SC, IPC) ^4^	25	MN	(TPC, SC, IPC) ^4^	10	Age, mitotic count, tumor grade, infiltrative growth
FI	(TPC, SC, IPC) ^4^	25	MI	(TPC, SC, IPC) ^4^	31	Age, mitotic count, tumor grade
FI	(TPC, SC, IPC) ^4^	25	MI	(TPC, SC, IPC) ^4^	30	Infiltrative growth
FI, FS	TPC	18	MI, MN	TPC	24	Age, mitotic count, tumor grade
FI, FS	TPC	18	MI, MN	TPC	23	Infiltrative growth
FI, FS	IPC	6	MI, MN	IPC	7	Age, mitotic count, tumor grade infiltrative growth

FI = female intact; FS = female spayed; MI = male intact; MN = male neutered; IPC = intraductal papillary carcinoma; SC = solid carcinoma; TPC = tubulopapillary carcinoma; ^1^ = guinea pigs with the sole presence of lobular hyperplasia; ^2^ = guinea pigs with a benign tumor and the possible additional presence of lobular hyperplasia; ^3^ = guinea pigs with a malignant tumor and the possible additional presence of lobular hyperplasia; ^4^ = all malignant tumors in more than 3 guinea pigs of this study; mitotic count = number of mitoses in 0.237 mm^2^; age = age of guinea pigs in years; tumor grade = I, II or III; infiltrative growth = infiltrative versus non-infiltrative tumor growth.

**Table 3:** Non-neoplastic mammary tissue and proliferative mammary lesions in 117 guinea pigs *.

Mammary Tissue and Lesions	Guinea Pigs		
	Numbers	FI/FS	MI/MN
Non-Neoplastic Lesions	50	49/1	0/0
Lobular hyperplasia with secretory activity	8	7/1	0/0
Lobular hyperplasia with secretory activity and a tumor	18	18/0	0/0
Lobular hyperplasia with fibrosis and a tumor	17	17/0	0/0
Lobular hyperplasia with secretory activity and fibrosis, and a tumor	7	7/0	0/0
**Benign Tumors**	**28**	**26**	**0/2**
(Simple) adenoma	20	20/0	0/0
Adenolipoma	3	3/0	0/0
Intraductal papillary adenoma	5	3/0	0/2
**Malignant Tumors**	**81**	**34/1**	**36/10**
(Simple) tubular carcinoma	3	1/0	2/0
(Simple) tubulopapillary carcinoma	41	17/1	19/4
(Simple) solid carcinoma	11	2/0	7/2
(Simple) tubulopapillary and (simple) solid carcinoma	1	0/0	1/0
Anaplastic carcinoma	1	0/0	1/0
Intraductal papillary carcinoma	13	6/0	3/4
Adenosquamous carcinoma	3	3/0	0/0
Malignant myoepithelioma	1	0/0	1/0
Carcinoma in (simple) adenoma	1	1/0	0/0
Adenoliposarcoma	1	1/0	0/0
Carcinosarcoma in adenolipoma	3	3/0	0/0
Metaplastic carcinoma	2	0/0	2/0
**Normal Mammary Tissue in Sections with a Proliferative Lesion**	**105**	**64/2**	**31/8**

* All 117 cases of this study had at least one proliferative mammary gland lesion; additional non-neoplastic mammary gland parenchyma was detected in 105 cases. Y = year; FI = female intact; FS = female spayed; MI = male intact; MN = male neutered; NA = not applicable; NK = not known.

**Table 4:** Statistical significant results.

Carcinomas in More Than 3 Animals (IPC, TPC, SC)				
Group 1	Group 2	Analysed Parameter(s)	Significance	Result
FI, FS (*n* = 26)	MI, MN (*n* = 41)	Mitotic count	*p* = 0.05	Higher in group 2
MI (*n* = 30)	MN (*n* = 10)	Infiltrative growth	*p* = 0.003	More frequent in group 2
FI (*n* = 25)	MN (*n* = 10)	Mitotic count	*p* = 0.047	Higher in group 2
FI (*n* = 25)	MN (*n* = 10)	Infiltrative growth	*p* = 0.010	More frequent in group 1
**Tubulopapillary carcinomas**				
**Group 1**	**Group 2**	**Analysed Parameter(s)**	**Significance**	**Result**
FI, FS (*n* = 18)	MI, MN (*n* = 24)	Mitotic count	*p* = 0.002	Higher in group 2

IPC = intraductal papillary carcinoma; TPC = tubulopapillary carcinoma; SC = solid carcinoma; FI = female intact; FS = female spayed; MI = male intact; MN = male neutered; Mitotic count = number of mitoses in 0.237 mm^2^; age of guinea pigs in years; tumor grade: I, II, or III; Infiltrative growth = analysis of infiltrative versus non-infiltrative tumor growth; *p* = *p*-value, *p* ≥ 0.05 = statistically significant.

## Data Availability

Further information on the data included in this study is available from the corresponding author upon reasonable request.

## Supplementary Materials

The following supporting information can be downloaded at: https://www.mdpi.com/article/10.3390/ani15111573/s1, Table S1. Immunolabelling of normal mammary tissue and proliferative mammary gland lesions of guinea pigs. Table S2. Non-neoplastic mammary tissue and proliferative mammary lesions in 117 guinea pigs including their ages. Figure S1. Histological features of mammary tissue in female guinea pigs. Figure S2. Microscopic findings of the mammilla. Figure S3. Histologic features of mammary tissue in male guinea pigs. Figure S4. Simple carcinomas. Infiltrative versus non-infiltrative growth and growth patterns.
