# Supplementary material for: Neoplastic and Non-Neoplastic Proliferative Mammary Gland Lesions in Female and Male Guinea Pigs: Histological and Immunohistochemical Characterization
Source: Animals (Basel). 2025 May 28;15(11):1573. doi: 10.3390/ani15111573 (PMC12153788; doi:10.3390/ani15111573)
Supplement: Supplementary file 1 [file animals-15-01573-s001.zip › animals-3602458-supplementary.pdf]

Table S1. Immunolabelling of normal mammary tissue and proliferative mammary gland lesions of guinea pigs.

Tissue or lesion	Marker (numbers of examined cases)
Female mammary gland	PanCK (16), p63 (18), CK 14 (5)
Male mammary gland	PanCK (3), p63 (7), CK 14 (1)
LH w. secr. activity and sebaceous diff.	PanCK (2), p63 (2), CK14 (2)
LH w. secr. activity	PanCK (6), p63 (6)
LH w. fibrosis	PanCK (4), p63 (3)
Tubular adenoma w. sebaceous diff.	PanCK (1), p63 (1), CK 14 (1)
Tubular adenoma	PanCK (2), P63 (4)
Intraductal papillary adenoma	P63 (1)
Adenolipoma	PanCK (1), p63 (1)
Intraductal papillary carcinoma	PanCK (1), p63 (4)
Tubular carcinoma	PanCK (1), p63 (1)
Tubulopapillary carcinoma	PanCK (4), p63 (6)
Solid carcinoma	PanCK (3), p63 (3)
Anaplastic carcinoma	PanCK (1), p63 (1)
Adenosquamous carcinoma	p63 (1)
Metaplastic carcinoma	p63 (2)
Malignant myoepithelioma	PanCK (1), p63 (1), CK14 (1)
CS w. sebaceous diff. in adenolipoma	PanCK (1), p63 (1), CK 14 (1), Vim (1), SMA (1)
CS in adenolipoma	PanCK (2), p63 (2), CK 14 (1), Vim (2), SMA (2)
Adenosarcoma	PanCK (1), p63 (1), CK14 (1), Vim (1), SMA (1)
Carcinoma in adenoma w. sebaceous diff.	p63 (1)

CK = cytokeratin; Vim = vimentin; SMA = α-smooth muscle actin; CS = carcinosarcoma; diff. = differentiation; LH = lobular hyperplasia; PanCK = cytokeratin AE1/AE3; secr. = secretory; w. = with

Table S2: Non-neoplastic mammary tissue and proliferative mammary lesions in 117 guinea pigs including their ages.

Mammary tissue and lesions	Guinea pigs					
	Numbers	Age(s) or Age range/ median (Y)	FI/FS	Age(s) or Age range/ median (Y)	MI/MN	Age(s) or Age range/ median (Y)
**Non-neoplastic lesions**	**50**	**1.00-9.00/3.63**	**49/1**	**1.00-9.00/3.63**	**0/0**	**NA**
LH w. secr. activity	8	1.00-6.25/2.96	7/1	1.00-6.25/2.96	0/0	NA
LH w. secr. activity and tumor	18	1.00-7.00/3.04	25/1	1.00-7.00/3.04	0/0	NA
LH w. fibrosis and tumor	17	2.00-7.00/4.00	17/0	2.00-7.00/4.00	0/0	NA
LH w. secr. activity, LH w. fibrosis and tumor	7	3.00-9.00/4.71	7/0	3.00-9.00/4.71	0/0	NA
**Benign tumors**	**28**	1.67-9.00/3.00	**26**	1.00-7.00/3.00	**0/2**	6.00; NK
(Simple) Adenoma	20	1.00-7.00/3.00	20	1.00-7.00/3.00	0/0	NA
Intraductal papillary adenoma	5	3.00-7.00/5.00	3/0	3.00; 3.00; 7.00	0/2	6.00; NK
Adenolipoma	3	3.17; 4.00; 7.00	3/0	3.17; 4.00; 7.00	0/0	NA
**Malignant tumors**	**81**	**1.50-9.00/4.00**	**34/1**	**1.50-9.00/4.00**	**36/10**	**1.58-6.00/3.67**
IPC	13	2.00-7.00/3.21	6/0	3.00-7.00/3.59	3/4	2.00-5.92/2.17
(Simple) T carcinoma	3	2.66; 4.00; 5.17	1/0	2.66	2/0	4.00; 5.17
(Simple) TP carcinoma	41	1.50-9.00/4.00	17/1	1.50-6.00/4.00	19/4	1.58-6.00/4.00
(Simple) S carcinoma	11	3.00-5.08/4.04	2/0	3.50; 3.83	7/2	3.00-5.08/4.04
(Simple) TP and (Simple) S carcinoma	1	4.00	0/0	NA	1/0	4.00
Adenosquamous carcinoma	3	3.00; 4.00; 9.00	3/0	3.00; 4.00; 9.00	0/0	NA
Anaplastic carcinoma	1	4.08	0/0	NA	1/0	4.08
Malignant myoepithelioma	1	3.25	0/0	NA	1/0	3.25
Metaplastic carcinoma	2	3.00; 3.00	0/0	NA	2/0	3.00; 3.00
CS in Adenolipoma	3	3.00; 3.83; 4.00	3/0	3.00; 3.83; 4.00	0/0	NA
Adenoliposarcoma	1	3.58	1/0	3.58	0/0	NA
Carcinoma in (simple) adenoma	1	4.00	1/0	4.00	0/0	NA
**Additional NMG**	**105**	**1.00-9.00/3.50**	**64/2**	**1.00-9.00/3.42**	**31/8**	**1.56-6.00/3.67**

All 117 cases of this study had at least one proliferative mammary gland lesion, additional non-neoplastic mammary gland parenchyma was detected in 105 cases. In 8 cases, lobular hyperplasia with secretory activity was the soley lesion. The concurrent presence of lobular hyperplasia with secretory activity and a tumor was observed in 18 cases, whereas lobular hyperplasia with fibrosis and the simultaneous presence of both types of lobular hyperplasia was always associated with a tumor. CS = Carcinosarcoma; IPC = intraductal papillary carcinoma; LH = Lobular hyperplasia; w. = with; secr. = secretory; S = solid; T = tubular; TP = tubulopapillary; NMG = normal mammary gland; Y = Year; FI = Female intact; FS = Female spayed; MI = Male intact; MN = Male neutered; NA = Not applicable; NK = Not known

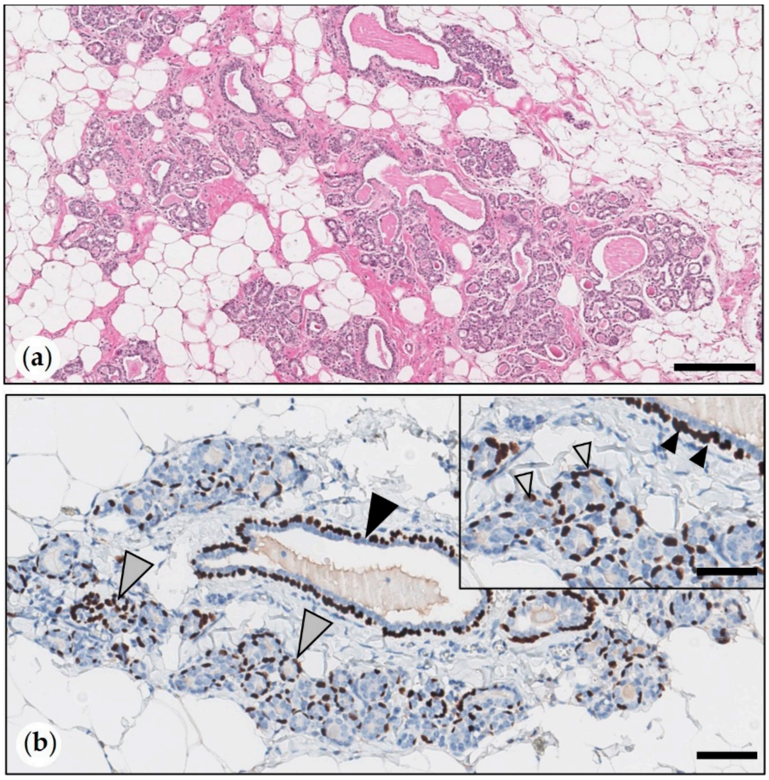

Figure S1. (**a-c**) Histological features of mammary tissue in female guinea pigs. (**a**) Terminal ductal lobular units (TDLUs) in the fat pad. Ducts contain secretory material. HE. Bar = 200 µm. (**b**) p63 immunolabelling: TDLU with selective labelling of myoepithelial cells (MECs) of the central duct (black arrowhead) and alveoli (grey arrowheads) by the nuclear marker p63. DAB. Bar = 100 µm. Inset: MECs of the central duct (black arrowheads) and alveoli (grey arrowheads) are shown in higher magnification. DAB. Bar = 50µm.

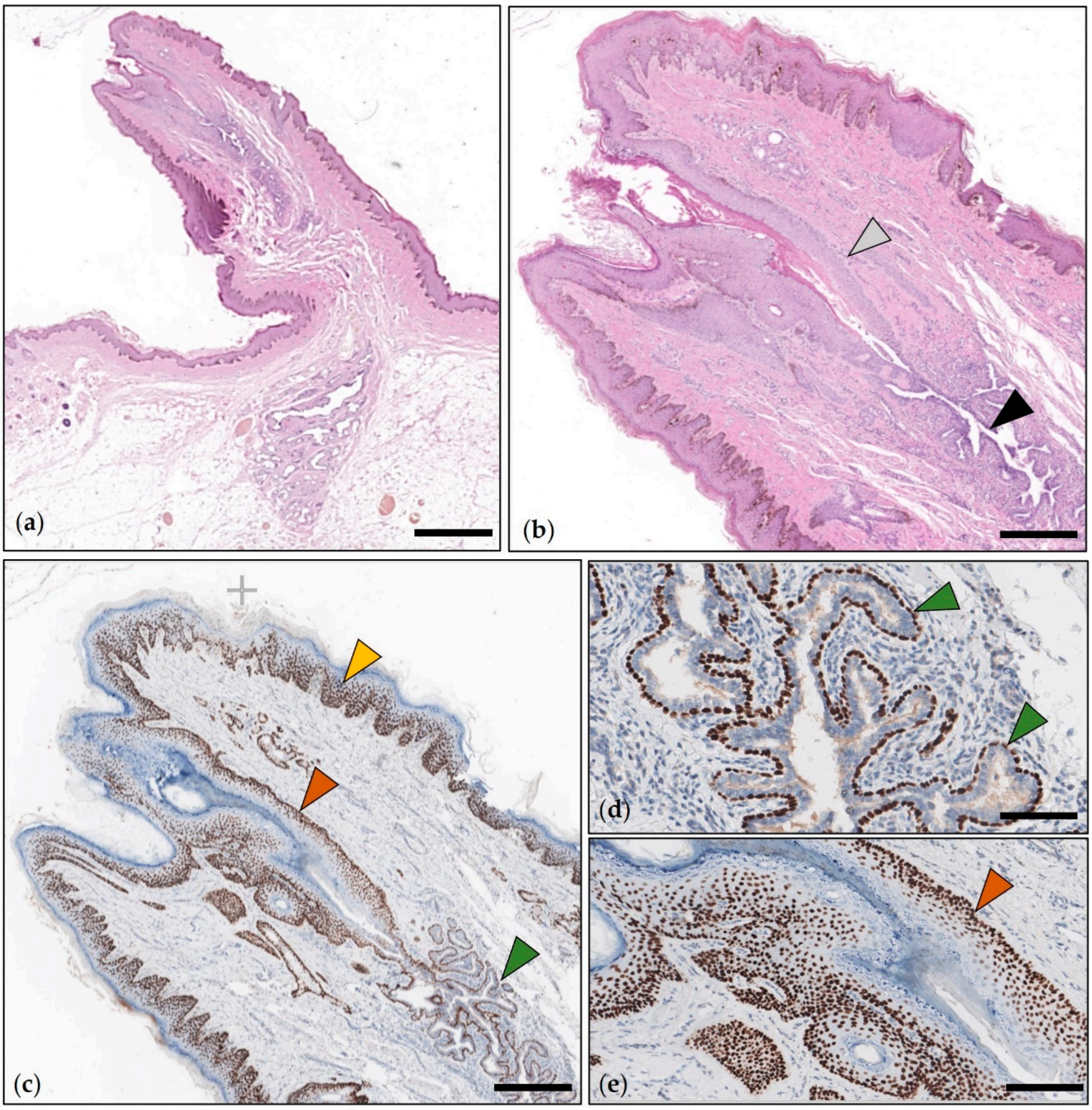

Figure S2. (**a-e**) Microscopic findings of the mammilla. (**a**) Mamilla with underlying lobular units of mammary parenchyma at an overview. HE. Bar = 1000 µm. (**b**) The mammilla is covered by keratinizing squamous epithelium and contains the teat duct (synonym: papillary duct). Its lower portion (black arrowhead) is lined by an inner layer of cuboidal epithelial cells and an underlying layer of suprabasal myoepithelial cells (MECs), its upper portion (grey arrowhead) is lined by squamous epithelium. HE. Bar = 300 µm. (**c-e**) Immunolabelling for p63: (**c**) p63 labelling highlights MECs lining the lower portion of the teat duct (green arrowhead) as well as basal and suprabasal keratinocytes in the squamous epithelium lining the upper portion of the teat duct (orange arrowhead) and the overlying skin (yellow arrowhead). DAB. Bar = 300 µm. (**d**) MECs lining the lower part of the teat duct (green arrowheads) are shown in detail. DAB. Bar = 100 µm. (**e**) In higher magnification depicted are MECs within the squamous epithelial portion of the teat duct (orange arrowhead). DAB. Bar = 100 µm.

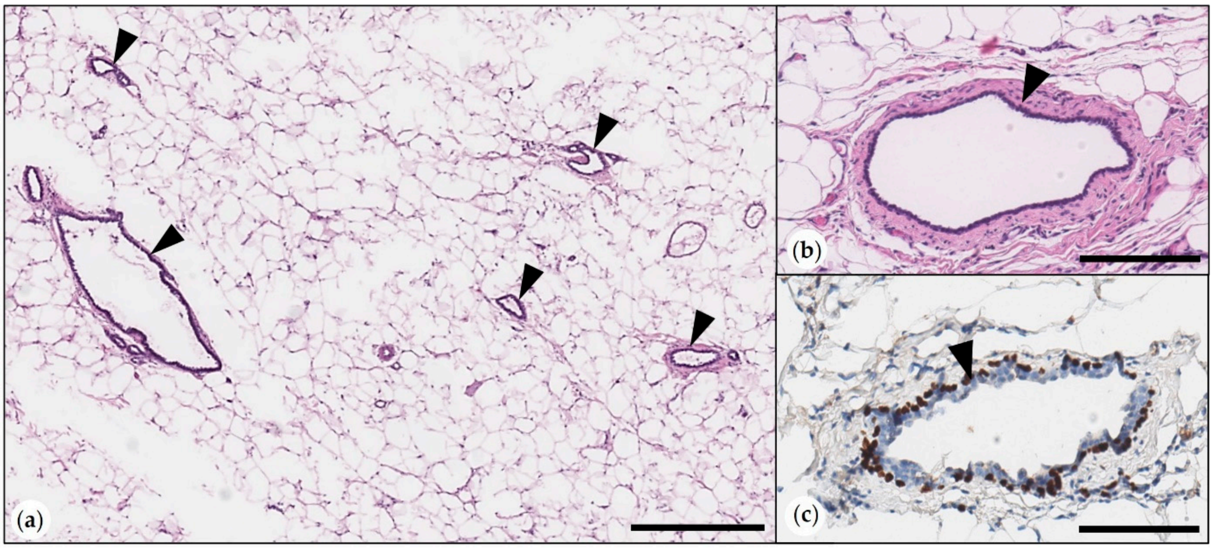

Figure S3. (**a-c**) Histologic features of mammary tissue in male guinea pigs. (**a**) The fat pad contains primitive ductal structures (arrowheads). HE. Bar = 500 μm. (**b**) Ductal structure in detail (arrowhead). HE. Bar = 100 μm. (**c**) P63 immunolabelling: Dutcal structure lined by inner immunonegative luminal epithelium and suprabasal p63 positive myoepithelial cells (arrowhead). DAB. Bar = 100 μm.

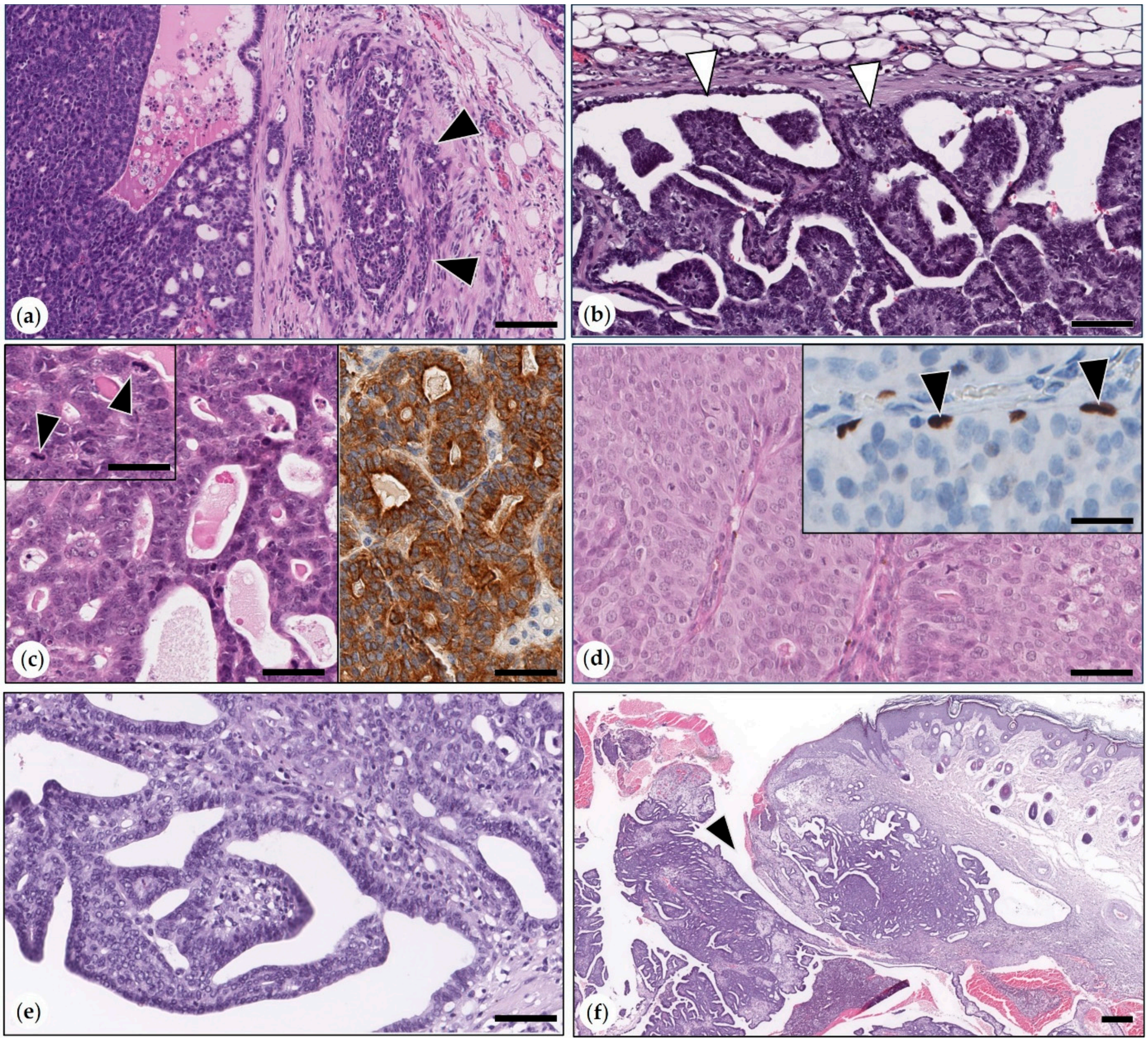

Figure S4. (a-f) Simple carcinoma. (a-b) Infiltrative versus non-infiltrative growth. (a) Tubulopapillary carcinoma with infiltrative tumor growth characterized by infiltration of adjacent tissue by tumor cells (arrowheads). HE. Bar = 150 µm. (b) Non-infiltrative tubulopapillary carcinoma. The tumor is relatively well demarcated from the surrounding tissue (arrowheads). HE. Bar = 70 µm. (c–e) Growth patterns of simple carcinomas. (c) Tubular carcinoma characterized by tubular structures that are lined by one to three layers of cuboidal to columnar tumor cells. In the depicted tumor, tubular structures contain proteinaceous fluid. HE. Bar = 50 µm. Small inset: Tumor cells have vesicular nuclei and one to several prominent nucleoli. Mitotic figures are indicated by arrowheads. HE. Bar = 30 µm. Large inset: The tubular morphology is discernable in the cytokeratin AE1/AE3 labelling. DAB. Bar = 50 µm. (d) Solid carcinoma composed of nests and trabecules of malignant epithelial cells separated by scant amount of stroma. HE. Bar = 100 µm. Inset: p63 immunolabelling shows a few remaining suprabasal myoepithelial cells (arrowheads) located at the margin of tumor cell nests, whereas tumor cells are immunonegative. DAB. Bar = 50 µm. (e) Tubulopapillary carcinoma characterized by the presence of variably-sized tubular structures with papillary infoldings supported by fibrovascular stroma. HE. Bar = 75 µm. (f) Tubulopapillary carcinoma with ulceration through the overlying skin (arrowhead). HE. Bar = 300 µm.
